# Causality Investigation between Gut Microbiota, Derived Metabolites, and Obstructive Sleep Apnea: A Bidirectional Mendelian Randomization Study

**DOI:** 10.3390/nu15214544

**Published:** 2023-10-26

**Authors:** Weiheng Yan, Miaomiao Jiang, Wen Hu, Xiaojun Zhan, Yifan Liu, Jiayi Zhou, Jie Ji, Shan Wang, Jun Tai

**Affiliations:** 1 Department of Otolaryngology, Head and Neck Surgery, Children’s Hospital Capital Institute of Pediatrics, Chinese Academy of Medical Sciences & Peking Union Medical College, Beijing 100020, China; yanwh@student.pumc.edu.cn (W.Y.); zjy20001005@163.com (J.Z.); 2 National Clinical Research Center for Mental Disorders (Peking University Sixth Hospital), NHC Key Laboratory of Mental Health (Peking University), Peking University Sixth Hospital, Peking University Institute of Mental Health, Beijing 100091, China; miao_mj@hsc.pku.edu.cn; 3 Department of Otolaryngology, Head and Neck Surgery, Children’s Hospital Capital Institute of Pediatrics, Beijing 100020, China; huwen2211@126.com (W.H.); cnczhan81@163.com (X.Z.); shoinlin@126.com (Y.L.); 4 Beijing Children’s Hospital, Capital Medical University, National Center for Children’s Health, Beijing 100045, China; joyjee0819@163.com; 5 Beijing Municipal Key Laboratory of Child Development and Nutriomics, Capital Institute of Pediatrics, Beijing 100020, China

**Keywords:** mendelian randomization, gut microbiota, obstructive sleep apnea, causal inference, inflammation, intestinal immunity

## Abstract

Various studies have highlighted the important associations between obstructive sleep apnea (OSA) and gut microbiota and related metabolites. Nevertheless, the establishment of causal relationships between these associations remains to be determined. Multiple mendelian randomization (MR) analyses were performed to genetically predict the causative impact of 196 gut microbiota and 83 metabolites on OSA. Two-sample MR was used to assess the potential association, and causality was evaluated using inverse variance weighted (IVW), MR-Egger, and weighted median (WM) methods. Multivariable MR (MVMR) was employed to ascertain the causal independence between gut microbiota and the metabolites linked to OSA. Additionally, Cochran’s Q test, the MR Egger intercept test and the MR Steiger test were used for the sensitivity analyses. The analysis of the 196 gut microbiota revealed that *genus*_*Ruminococcaceae* (*UCG009*) (P_IVW_ = 0.010) and *genus*_*Subdoligranulum* (P_IVW_ = 0.041) were associated with an increased risk of OSA onset. Conversely, *Family*_*Ruminococcaceae* (P_IVW_ = 0.030), *genus*_*Coprococcus2* (P_WM_ = 0.025), *genus*_*Eggerthella* (P_IVW_ = 0.011), and *genus*_*Eubacterium* (*xylanophilum*_*group*) (P_IVW_ = 0.001) were negatively related to the risk of OSA. Among the 83 metabolites evaluated, 3-dehydrocarnitine, epiandrosterone sulfate, and leucine were determined to be potential independent risk factors associated with OSA. Moreover, the reverse MR analysis demonstrated a suggestive association between OSA exposure and six microbiota taxa. This study offers compelling evidence regarding the potential beneficial or detrimental causative impact of the gut microbiota and its associated metabolites on OSA risk, thereby providing new insights into the mechanisms of gut microbiome-mediated OSA development.

## 1. Introduction

Obstructive sleep apnea (OSA) is a commonly occurring sleep disorder distinguished by recurring instances of upper airway constriction or collapse during sleep, leading to intermittent hypoxia (IH) and disruptions in sleep patterns [1]. OSA can lead to a wide range of complications, including cognitive disorders, cardiovascular diseases, metabolic diseases, and other systemic conditions [2,3,4]. The onset age of OSA spans the entire lifespan, from infancy to middle and old age, imposing a significant medical burden [5,6]. The etiology of OSA is complex. Alterations in the craniofacial structure, enlarged tonsils, edema of the upper respiratory tract, reduced lung volume, and obesity are immediate causes of OSA [7,8]. However, the underlying etiologies of these immediate factors remain largely unknown.

The gut microbiome undergoes substantial modifications in both adolescent and adult patients diagnosed with OSA [9,10]. Several studies have identified a variety of significant imbalances in patients with OSA, including *Clostridia*, *Ruminococcus*, *Faecalibacterium*, and numerous bacteria that produce short-chain fatty acids (SCFAs) [11,12,13,14]. Thus, new strategies involving prebiotics, probiotics, and SCFAs are expected to emerge for targeting OSA-mediated dysbiosis in the gut microbiome [15]. Previous studies have observed that OSA causes changes in a variety of serum metabolites, including porphyrins, glycerophospholipids (GPL), fatty acids, eicosanoids, and amino acids. These metabolites are mostly associated with oxidative stress [16]. Additionally, reduced acetate and butyrate levels were observed in individuals with OSA-associated hypertension. These disruptions in the SCFA metabolism are considered to be an integral part of the pathogenesis of OSA [17]. Hypoxia-induced oxidative stress may lead to metabolic changes in OSA patients. A study conducted in IH animal models of OSA have found that IH leads to reduced levels of metabolites, including pyruvate, citric acid, succinic acid, and acetoacetic acid, as well as increased levels of oxides such as trimethylamine oxide (TMAO) in urine [18]. Similarly, a study conducted in human subjects observed elevated levels of branched-chain amino acids (BCAAs) such as leucine, isoleucine, and valine [19]. It has been found that chronic IH regulates gut microbes (e.g., *Clostridium*, *Lactococcus*, and *Bifidobacterium*) and the abundance of important functional metabolites (such as free fatty acids and bile acids), thereby promoting the occurrence of lipid metabolism disorders [14]. Furthermore, supplementation with probiotics and key short-chain fatty acids effectively treats hypertension associated with IH [20]. However, the animal model for IH has limitations, as it cannot accurately simulate sleep fragmentation and hypercapnia caused by OSA [21]. Additionally, in most observational studies on OSA, the association between OSA and gut microbiota/metabolites is susceptible to confounding factors. These limitations of current research methods hinder the establishment of causal relationships between gut microbiota/metabolites, and OSA [9,12,22]. 

Mendelian randomization (MR) is a novel method for investigating the causal relationship between gut microbiota and OSA. In the MR analysis, genetic variants are used as proxies for prospective exposure factors through a succession of algorithms to assess the causal effect of exposure on outcomes [23]. MR analysis can maximally eliminate the interference of common environmental confounding factors, avoiding confusion regarding the chronological order of exposure and outcome. In this study, a bidirectional two-sample MR was used to determine the causal relationship between 196 gut microbiota groups, 83 types of microbiota-derived metabolites, and OSA risk. In addition, we aimed to provide new evidence and insights regarding the role of gut microbiota and derived metabolites in the etiology and pathological process of OSA from a genetic perspective.

## 2. Materials and Methods

### 2.1. Study Design Overview and Data Sources

**Figure 1:**
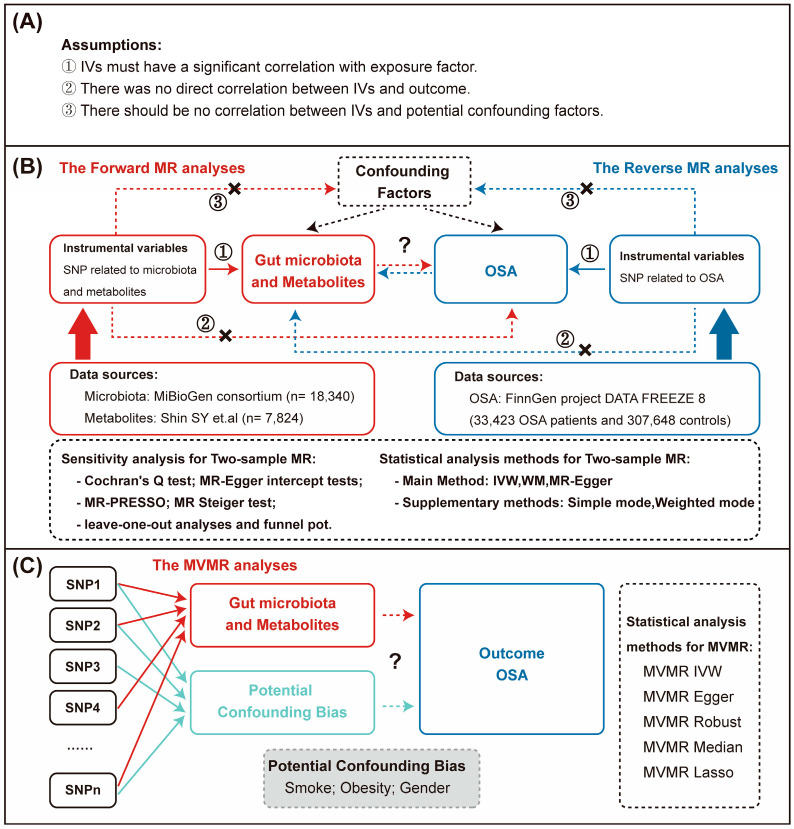
The outline of the methodology. (**A**) The assumptions for MR analysis. (**B**) The flow of two-sample MR analysis [26]. (**C**) The flow of MVMR analysis. IVs must have a significant correlation with exposure factor (assumption 1). There was no direct correlation between IVs and outcome (assumption 2). There should be no correlation between IVs and potential confounding factors (assumption 3). Jiayi Zhou IVW, inverse-variance weighted; WM, weighted median; MR, Mendelian randomization; MR-PRESSO, MR pleiotropy residual sum and outlier; OSA, obstructive sleep apnea; IVs, instrumental variables; MVMR, multivariate Mendelian randomization.

We first used two-sample MR to comprehensively assess the bidirectional causal relationship between the gut microbiota and its derived metabolites and OSA, as well as to screen potential microbiota and metabolites related to OSA. Subsequently, multivariate mendelian randomization (MVMR) analysis was employed to evaluate whether these potential microbiota and metabolites had an independent effect on the development of OSA. The outline of the methodology is represented in Figure 1. 

All genome-wide association study (GWAS) data included in this study were limited to populations of European descent. The FinnGen project (DATA FREEZE 8, https://www.finngen.fi/en, accessed on 23 March 2023) was used to obtain the GWAS summary statistics for OSA. The FinnGen Project is a large genetic research program that aims to explore the relationship between genomic information and health characteristics in Finnish populations (Europeans). The R8 data include GWAS information and health characteristic records for 342,499 individuals. GWAS data for OSA were obtained by submitting a request to the investigators of the FinnGen study for approval. The dataset includes 33,423 patients with OSA and 307,648 control individuals [24]. Summary statistics for the gut microbiota were derived from the largest gut microbiota GWAS project; up to 18,340 individuals were included in this project [25].

We included the largest collection of GWAS summary data on metabolomics in a European population to date [26]. This project comprised GWAS data for more than 400 metabolites from 7824 samples. We used the Human Metabolome Database (HMDB, https://hmdb.ca/, accessed on 10 May 2023, version 5.0) to screen for metabolites associated with the gut microbiota. The HMDB database contains metabolite information from different sources, such as blood, stool, and urine of human subjects [27]. We manually searched the database to extract all fecal metabolites (download link to list of metabolites: https://hmdb.ca/downloads, accessed on 10 May 2023). We then annotated the metabolites from the metabolomics project [26] based on fecal metabolites of HMDB. In total, 83 gut microbiota-derived metabolites were selected.

In order to mitigate potential gender bias, these three datasets used in our study were carefully selected to include individuals of both genders, thereby ensuring a balanced representation and minimizing the impact of gender-related confounding factors on our analysis.

### 2.2. Selection of IVs

As reported in a previous study, a genome-wide significance level (*p* < 1 × 10^−5^) was used as the global IVs screening criterion to obtain sufficient IVs for subsequent analyses [28]. The 1000 Genomes European Sample Data were employed as the criteria to remove linkage disequilibrium. A minor allele frequency threshold of 0.3 was permitted for the palindromic single nucleotide polymorphisms (SNPs). Additionally, the clumping threshold was set to R^2^ < 0.001, and the clumping window size was defined as  10,000 kb. 

### 2.3. Two-Sample MR Analysis Methods

All analyses were performed using the R software (version 4.1.1). MR analyses were conducted using the TwoSampleMR package (version 0.5.6) [29]. The main analysis was conducted using the inverse variance weighted (IVW), MR-Egger, and weighted median (WM). At least one of the three main methods suggested a significant causal relationship. Additionally, simple and weighted-mode methods were employed as Supplementary Analytical Methods (see Additional File S1: Supplementary Methods). The direction of the MR analysis results (beta values) was consistent across all five methods.

### 2.4. MVMR Analysis Methods

To consider the potential for genetic confounding, traits such as obesity, sex, and smoking were examined in multivariable MR (MVMR) analysis. In simple terms, MVMR disentangles the direct influence of each risk factor on the outcome event by incorporating the genetic variation of each risk factor into the same model [30]. In our study, we constructed an MVMR analysis model by associating potentially associated microbiota/metabolites with each common OSA risk factor, to assess whether microbiota and metabolites are still directly associated with changes in OSA risk while taking these common risk factors into account. MVMR-Robust, MVMR-IVW, MVMR-Egger, MVMR-Median, and the Least absolute shrinkage and selection operator (Lasso) were used to determine the independent effects between exposure and outcome [31]. When at least one MVMR method provided significant evidence of causality, the causal relationship was considered robust (see Additional File S1: Supplementary Methods). 

### 2.5. Pleiotropy and Sensitivity Analysis

Cochran’s IVW Q statistics were used to calculate potential heterogeneity among the IVs [32]. Additionally, a “leave-one-out” evaluation was performed to identify potential heterogeneous SNPs by excluding each IV SNP. The intercept from the MR-Egger regression was employed to assess the horizontal pleiotropy of the IVs. The MR-pleiotropy residual sum and outlier (MR-PRESSO) method was used to supplement the assessment of the pleiotropy of instrumental SNPs and to screen for possible outlier SNPs [33]. The MR-Steiger test was used to determine the probable direction of this relationship [34]. The Bonferroni correction was applied to adjust the *p*-values, with a significance threshold set at 0.00017 (0.05/279) to determine strong evidence of a causal association.

### 2.6. Enrichment Analysis of Microbiota-Derived Metabolites

The HMDB database was used for the classification and molecular annotation of metabolites [27]. The MetaboAnalyst (version 5.0) platform was employed to analyze and interpret the functions of the metabolites [35]. 

## 3. Results

Our research included 196 known taxa (Additional File S2: Table S1) and 83 microbiota-derived metabolites (Additional File S2: Table S2) after excluding undetermined microbiota taxa. The global F-value of the IVs ranged from 17.75 to 760.95, which helped to avoid the confounding bias associated with weak IVs. Details of all IVs included in this study are presented in the Supplementary Materials (Additional File S2: Table S3, Table S9, Table S15 and Table S21).

### 3.1. Causal Relationship between OSA and Gut Microbiota in Two-Sample MR Analysis

**Figure 2:**
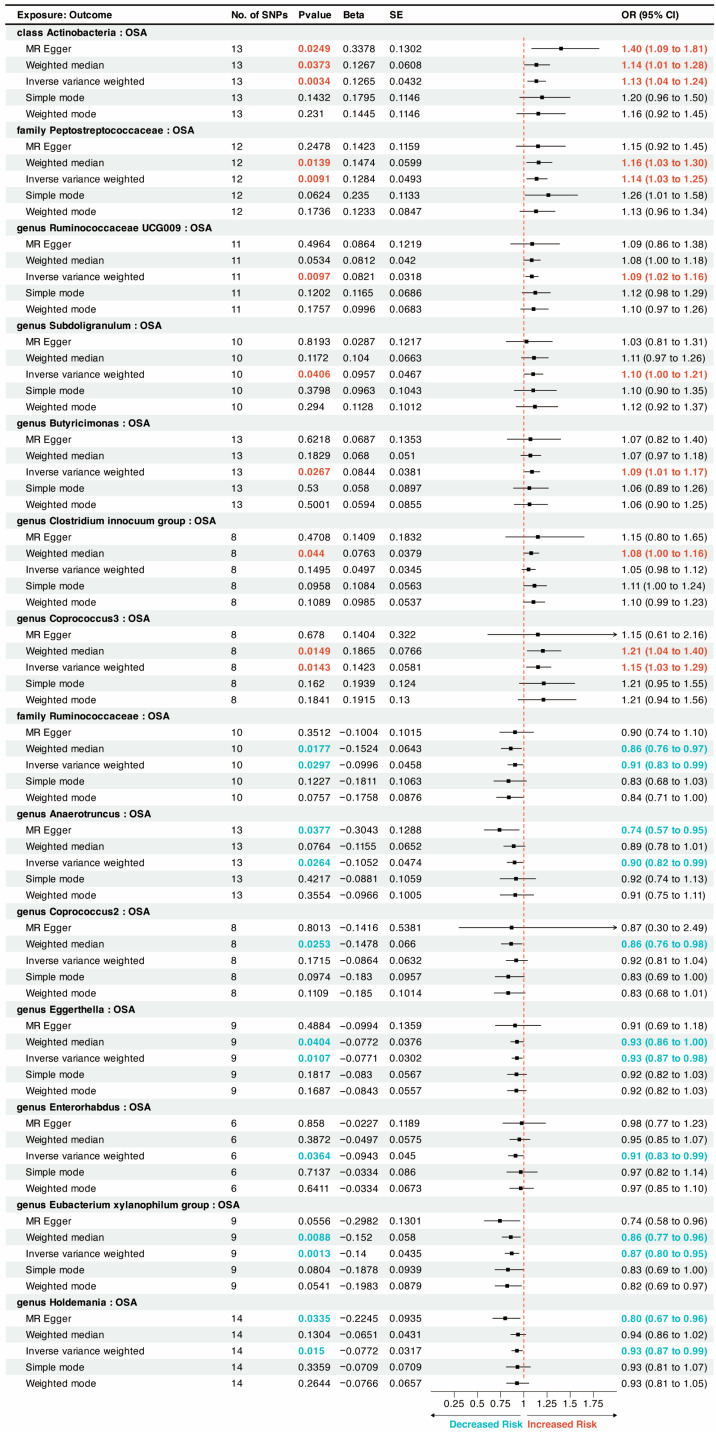
A forest plot of the potential causal relationship between gut microbiota and OSA. CI, confidence interval; OR, odds ratio; OSA, obstructive sleep apnea; SNP, single-nucleotide polymorphism; SE, standard error.

Fourteen microbial taxa were associated with OSA. Among them, seven microbiota taxa were associated with an increased risk of OSA, including *class*_*Actinobacteria* (OR = 1.13, P_IVW_ = 0.0034), *family*_*Peptostreptococcaceae* (OR = 1.14, P_IVW_ = 0.0091), *genus*_*Ruminococcaceae*_*UCG009* (OR = 1.09, P_IVW_ = 0.0097), *genus*_*Subdoligranulum* (OR = 1.10, P_IVW_ = 0.0406), *genus*_*Butyricimonas* (OR = 1.09, P_IVW_ = 0.0267), *genus*_*Clostridium* (*innocuum group*) (OR = 1.08, P_WM_ = 0.044), and *genus*_*Coprococcus3* (OR = 1.15, P_IVW_ = 0.0143) (Figure 2).

The results showed that seven microbiota taxa was associated with a decreased risk of OSA (Figure 2). The detailed results can be viewed in Additional File S2: Table S4. They were as follows: *family*_*Ruminococcaceae* (OR = 0.91, P_IVW_ = 0.0297), *genus*_*Anaerotruncus* (OR = 0.90, P_IVW_ = 0.0264), *genus*_*Coprococcus2* (OR = 0.86, P_WM_ = 0.0253), *genus*_*Eggerthella* (OR = 0.93, P_IVW_ = 0.0107), *genus*_*Enterorhabdus* (OR = 0.91, P_IVW_ = 0.0364), *genus*_*Eubacterium* (*xylanophilum group*) (OR = 0.87, P_IVW_ = 0.0013), and *genus*_*Holdemania* (OR = 0.93, P_IVW_ = 0.0150).

**Figure 3:**
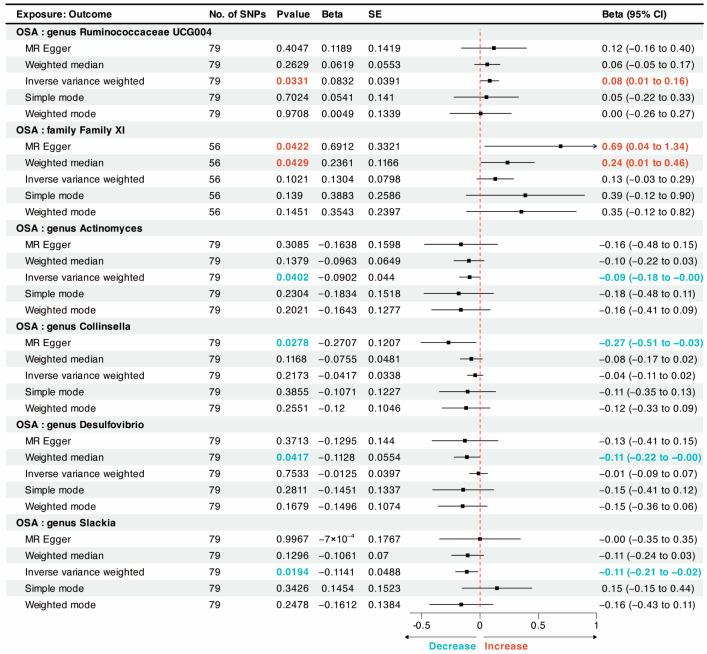
A forest plot of the potential causal relationship between OSA and gut microbiota. CI, confidence interval; OR, odds ratio; OSA, obstructive sleep apnea; SNP, single nucleotide polymorphism; SE, standard error.

The reverse MR analysis was conducted to investigate the putative causal effects of OSA and all 196 microbiota taxonomies (Additional File S2: Table S10). The results showed a nominally causal effect OSA on *genus*_*Ruminococcaceae* (*UCG004*) (beta = 0.08, P_IVW_ = 0.0331), *family*_*Family_XI* (beta = 0.24, P_WM_ = 0.0429), *genus*_*Actinomyces* (beta = –0.09, P_IVW_ = 0.0402), *genus*_*Collinsella* (beta = –0.27, P_Egger_ = 0.0278), *genus*_*Desulfovibrio* (beta = –0.11, P_WM_ = 0.0417), and *genus*_*Slackia* (beta = –0.11, P_IVW_ = 0.0194) (Figure 3). 

### 3.2. Causal Relationship between OSA and Gut Microbiota Metabolites in Two-Sample MR Analysis 

**Figure 4:**
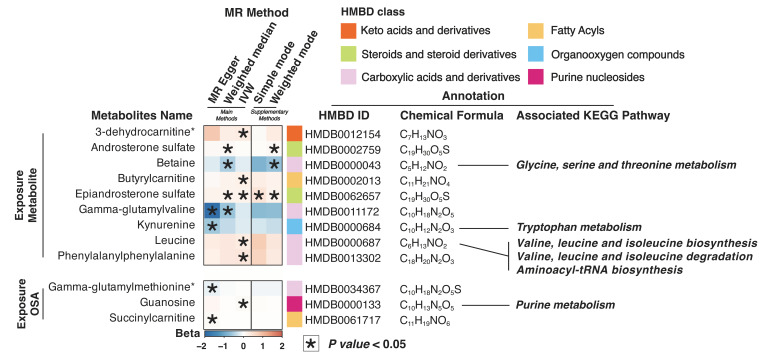
Heatmap plot of the potential causal relationship between OSA and metabolite. For upper part, exposure: outcome (metabolite: OSA); for lower part, exposure: outcome (OSA: metabolite). Enrichment map of HMBD analysis for the metabolites. * *p* < 0.05. OSA, obstructive sleep apnea; IVW, inverse-variance weighted.

The bidirectional causal relationships between the 83 metabolites and OSA were evaluated (Additional File S2: Table S16). Among them, betaine (OR = 0.60, P_WM_ = 0.0191), gamma-glutamylvaline (γ-EV) (OR = 0.55, P_WM_ = 0.0316), and kynurenine (OR = 0.56, P_Egger_ = 0.0486) were found to have potential protective effects against OSA (Figure 4). In addition, 3-dehydrocarnitine, androsterone sulfate, butyrylcarnitine, epiandrosterone sulfate, leucine, and phenylalanylphenylalanine were potentially associated with an increased risk of OSA (OR > 1, *p* < 0.05).

The reverse MR analysis investigating suggestive association between exposure to OSA and three metabolites (Additional File S2: Table S22), including gamma-glutamylmethionine (beta = −0.12, P_Egger_ = 0.0420), guanosine (beta = 0.06, P_IVW_ = 0.0166), and succinylcarnitine (beta = 0.06, P_Egger_ = 0.0364) (Figure 4). 

Enrichment analysis revealed that the metabolites causally related to OSA were mainly associated with key metabolic pathways, including glycine, serine, and threonine metabolism; tryptophan metabolism; valine, leucine, and isoleucine biosynthesis; aminoacyl-tRNA biosynthesis; and purine metabolism in the KEGG pathway. 

### 3.3. Sensitivity Analysis

When assessing the causal relationship between betaine, leucine, and OSA, we observed heterogeneity among the IVs (IVW Cochrane Q test: *p*-value < 0.05); however, no horizontal pleiotropy were found. In addition, for the other potential causal associations, the Cochrane Q test showed no heterogeneity among the IVs, and the MR-Egger regression demonstrated no horizontal pleiotropy (Additional File S2: Tables S3–S26). The leave-one-out analysis and funnel plot confirmed the robustness of the MR results (Additional File S3: Supplementary Figures). The MR Steiger test provided further support for our conclusions regarding all 14 potential causal relationships between the gut microbiota and OSA (Additional File S2: Tables S3–S26).

### 3.4. MVMR Analysis for OSA and Gut Microbiota/Metabolites

**Figure 5:**
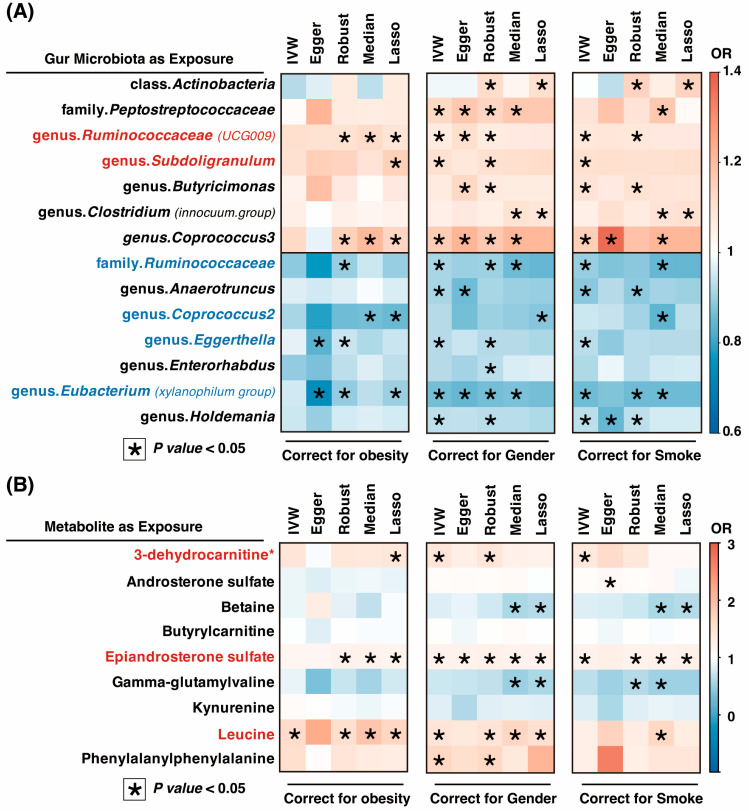
MVMR associations of OSA with gut microbiota and metabolites. For each MVMR analysis, we added each genetic confounding separately. (**A**) The direct causality between gut microbiota and OSA. (**B**) The direct causality between metabolites and OSA. Lasso, least absolute selection and shrinkage operator; IVW, inverse-variance weighted; MVMR, multivariate Mendelian randomization; Median, weighted-median; OR, odds ratio; OSA, obstructive sleep apnea. * *p* < 0.05.

MVMR analysis revealed that some gut microbiota and metabolites were associated with OSA (Figure 5). For example, when adjusted for obesity, *family*_*Ruminococcaceae* (adjusted OR = 0.90, *P*_Robust_ = 0.0426), *genus*_*Coprococcus2* (adjusted OR = 0.86, *P*_LASSO_ = 0.0031), *genus*_*Eggerthella* (adjusted OR = 0.94, *P*_Robust_ = 0.0237), and *genus*_*Eubacterium* (*xylanophilum group*) (adjusted OR = 0.89, *P*_Robust_ = 0.0223) demonstrated negative correlations with the occurrence of OSA. Even after adjusting for gender and smoke, these associations were also robust (Figure 5A and Additional File S2: Table S27). The *genus*_*Ruminococcaceae* (*UCG009*) and *genus*_*Subdoligranulum* were found to be associated with an increased risk of OSA.

Alternatively, when metabolites were used as exposures, 3-dehydrocarnitine, epiandrosterone sulfate, and leucine were found to be potentially independent risk factors for the development of OSA (Figure 5B and Additional File S2: Table S28). When adjusted for obesity, the associations were as follows: 3-dehydrocarnitine on OSA (adjusted OR = 1.40, *P*_Lasso_ = 0.0231), epiandrosterone sulfate on OSA (adjusted OR = 1.19, *P*_Robust_ = 1.44 × 10^−5^), and leucine on OSA (adjusted OR = 1.70, *P*_Robust_ = 0.0009). Even after adjusting for gender and smoking, these associations were also robust. 

## 4. Discussion

This study is the first to assess the bidirectional causal relationship between 196 gut microbiota- and 83 microbiota-derived metabolites and OSA risk. The *genus_Ruminococcaceae* (*UCG009*) and *genus_Subdoligranulum* might be risk factors for OSA onset. Moreover, the levels of the *family_Ruminococcaceae*, *genus-Coprococcus2*, *genus_Eggerthella*, and *genus_Eubacterium* (*xylanophilum group*) were negatively correlated with the risk of OSA. The MVMR analysis revealed that some metabolites (3-dehydrocarnitine, epiandrosterone sulfate, and leucine) were potential independent risk factors for OSA development. Our findings highlight the potential impact of disturbances in the gut microenvironment, alterations in derived metabolites, and increased systemic pro-inflammatory responses on OSA. These findings provide important information regarding the potential gut-microbiome-related pathogenesis and targeted treatment strategies for OSA.

### 4.1. Amino Acid and Lipid Metabolism Disorders Associated with OSA

The study demonstrated that gut microbiota-derived metabolites, particularly leucine and 3-dehydrocarnitine, were associated with an increased risk of OSA, and this causal relationship remained robust even after adjusting for obesity, sex, and smoking. Notably, elevated leucine and isoleucine levels were observed in children diagnosed with OSA but were not associated with obesity [36], and circulating isoleucine levels were associated with the OSA wakefulness index and sleep fragmentation. Conversely, randomized controlled trials suggested a decrease in leucine levels in individuals with OSA treated with continuous positive airway pressure [37]. The presence of OSA is a risk factor for type 2 diabetes and significantly increases the risk of diabetic nephropathy [38]. Meta-analysis has shown that elevated leucine is associated with an increased risk of developing type 2 diabetes [39]. In addition, lipid metabolism disorders in OSA are closely associated with cardiovascular diseases and diabetes [40], and 3-dehydrocarnitine (a carnitine precursor) and butyrylcarnitine (a short-chain acylcarnitine) are involved in fatty acid metabolism [41]. Moreover, patients with severe sleep apnea showed a significant increase in the levels of free fatty acids throughout the sleep cycle compared to controls [42], and a plasma metabolomic study revealed that butyrylcarnitine levels were associated with steatosis [43]. Furthermore, a previous MR study reported an increased risk of polycystic ovary syndrome (PCOS) with genetically predicted levels of 3-dehydrocarnitine [44], and an observational study suggested that the prevalence of OSA was higher in patients with PCOS. That study also found that androgen metabolites, including epiandrosterone sulfate and androsterone sulfate, were related to OSA. Interestingly, increased free testosterone levels were found to be associated with a high risk of PCOS combined with OSA [45]. Microorganisms can metabolize carnitine to betaine, and betaine supplementation can enhance the antioxidant capacity and protect the central nervous system [46,47]. Notably, in the present study, betaine was associated with a reduced risk of OSA. Animal experiments have shown that transplantation of gut microbiota leads to changes in metabolism and sleep status [48]. Based on these findings, further exploration is needed to determine whether microbial-targeted modifications aimed at reducing leucine levels and supplement betaine may be an effective intervention for OSA.

### 4.2. Inflammation and OSA

OSA patients commonly experience IH and sleep disruption, which can lead to oxidative stress and inflammatory responses in the brain [49]. In this study, gamma-glutamylmethionine (γ-EV) was found to have a potential protective effect against OSA. A previous study has shown that activation of the calcium-sensing receptor (CaSR) by α-EVs has an anti-inflammatory effect on mouse adipocytes, resulting in maintaining intestinal homeostasis [50]. The fragmented sleep experienced by individuals with OSA can contribute to dysbiosis of the gut microbiota and disruption of intestinal homeostasis, consequently leading to the occurrence of gastrointestinal comorbidities such as gastroesophageal reflux disease [51]. The γ-Glutamyl peptide, which is present in beans, garlic, and onions, among other foods, is known for its potential as an anti-inflammatory peptide. This is attributed to its ability to activate CaSR. Furthermore, studies conducted in vitro have demonstrated the anti-inflammatory properties of γ-EV on gastrointestinal inflammation and vascular endothelial cells [52]. IH elicits sympathetic nervous system activation and provokes oxidative stress, thereby initiating a cascade of systemic inflammation [53]. Therefore, it is speculated that γ-EV can reduce the systemic multi-system damage caused by OSA through its anti-inflammatory effect. Moreover, a cohort study has demonstrated the negative correlation between γ-EV and chronic kidney disease through metabolomics [54]. Collectively, γ-EV may have health benefits through its anti-inflammatory and anti-obesity properties, consistent with the observations in the present study.

In addition, our study establishes a link between SCFA-producing bacteria and a reduced risk of OSA, such as *family*_*Ruminococcaceae*. *Ruminococcaceae* is one of the primary bacteria responsible for producing SCFAs, and variations in its abundance can influence SCFA secretion [55,56]. SCFAs derived from *Ruminococcaceae* have been shown to reduce intestinal inflammation and promote stem cell differentiation and repair [57]. Hence, it is plausible that the protective effects of *Ruminococcaceae* against OSA are partially attributable to SCFA production. SCFAs, primarily acetate, propionic acid, and butyric acid, are the primary end products of human gut microbiota metabolism [58]. In OSA patients, both gut microbial diversity and the ratio of firmicutes to Bacteroides are reduced [59]. Moreover, CIH has been found to alter the distribution of intestinal microbiota in mice [14]. SCFAs are among the most important microbiota-derived metabolites. Several animal and clinical studies have found reduced SCFA levels and diminished abundance of SCFA-producing bacteria in individuals with OSA or IH animal models [12,60,61]. Notably, SCFAs, mainly acetate, can promote the differentiation and secretion of intestinal epithelial goblet cells [62], thereby improving the tight functional connection of intestinal epithelial cells, enhancing the intestinal epithelial immune defense barrier function [63,64] and synergistically inhibiting the formation of harmful bacteria and their metabolites [65]. Furthermore, acetate and butyrate exert anti-inflammatory effects by restoring the Th17/Treg imbalance [66]. In individuals with OSA, a decrease in SCFA-producing bacteria, an imbalance in effector helper T cells (Th cells), and an increase in the corresponding inflammatory cytokines were also found [17]. Therefore, SCFA supplementation and fecal microbiota transplantation are expected to become effective treatments for OSA [15]. As important SCFA producers, *Ruminococcaceae* is also associated with OSA comorbidities. *Ruminococcaceae* (*UCG013*) has been shown to promote obesity resistance in mice [67]. *Ruminococcaceae* (*UCG010*) was significantly reduced in the gut of hypertensive patients [68]. However, to date, no clinical trials have described the beneficial effects of probiotic supplementation on OSA prevention. As such, further studies are needed to evaluate whether probiotics and SCFAs are promising novel strategies against OSA-mediated dysbiosis. 

### 4.3. Intestinal Microenvironment Disorders and OSA

In the present study, several gut microbial taxa were identified as potentially protective against OSA, with a significant proportion belonging to the *family*_*Ruminococcaceae*. *Ruminococcaceae* are of great value in maintaining intestinal homeostasis and the stability of the gut microbiota [69]. A study found a significant reduction in *Ruminococcaceae* abundance among patients with mild, moderate, and severe OSA (*n* = 113) [12]. These findings are consistent with our results, further supporting the potential protective effect of *Ruminococcaceae* abundance against OSA. 

What is clearly known is that most bacteria in the *Ruminococcaceae* family are involved in bile acid (BA) metabolism. Recent studies have indicated that BA metabolism is closely linked to sleep. Specifically, BA metabolites influence sleep regulatory centers and circadian rhythms [70], affecting human sleep quality and health. Animal experiments conducted by Ferrell et al. [71] demonstrated that even short-term (no more than five days) circadian disruption substantially altered the expression of hepatic clock genes and BA metabolism. In addition, Kanemitsu et al. [72] found that specific BAs block the activation of circadian transcription factors and the nuclear receptor peroxisome proliferator-activated receptor y. However, there is no unified theory linking IH to BA regulation in humans or animals. It has only been observed in animal models that CIH disrupts BA metabolism [73]. In our study, we also identified the *genus*_*Eubacterium* (*xylanophilum group*) and two genera (*Anaerotruncus* and *Coprococcus2*) belonging to *genus*_*Ruminococcaceae* as protective factors against OSA. The *genus*_*Eubacterium* is significantly associated with secondary BA synthesis [74]. Our study provides preliminary evidence supporting the possible protective role of *Ruminococcaceae* and BA metabolism in the pathogenesis of OSA. However, further larger-scale prospective studies are needed to reveal the possible role of the gut microbiota belonging to *Ruminococcaceae* in sleep-disordered breathing.

### 4.4. Strengths and Weaknesses

This study has several strengths. First, this study utilized the largest and most recent GWAS data collection for OSA, comprising 33,423 patients and 307,648 controls. Second, we separately examined the causal relationships between the gut microbiota or metabolites and OSA and identified three microbe-derived metabolites that were associated with an increased risk of OSA for the first time. Third, this is the first MR study to evaluate the causal relationship between OSA and gut microbiota. It utilized various complementary MR methods and confounding bias correction for recognized risk factors for OSA. As a result, this study provides independent evidence of the association between gut microbiota and OSA risk. 

However, this study had several limitations. First, the GWAS included in this study was based on European populations. Future research could include populations with diverse characteristics, which may increase the representation of the present findings across different populations. Second, the GWAS data for gut microbiota included in this study did not contain detailed species-level information. The analysis was only performed at the genus level but not at a more specialized level, such as species or strain levels. Future studies exploring the relationship between specific intestinal bacteria and OSA at the species level could provide more theoretical support for the involvement of the microbiome in the pathophysiological mechanisms of OSA.

### 4.5. Future Directions

There are several avenues for future research that can expand upon and further elucidate the scope of the relationship between gut microbiota and OSA. Tracking changes in gut microbiota composition and their association with OSA development and clinical outcomes would provide valuable insights into the dynamic nature of this relationship. Additionally, further mechanistic investigations are warranted to unravel the underlying mechanisms through which specific microbial taxa and their derived metabolites modulate the occurrence of sleep apnea events. Exploring the interactions between these microbial communities and key factors such as the immune system, inflammatory responses, and the gut–brain axis could shed light on the potential mechanisms driving OSA. Interventions targeting the gut microbiota, such as dietary modifications, probiotic supplementation, or fecal microbiota transplantation, could be explored to modify the composition and metabolite profiles of microbial communities, thus improving symptoms and sleep quality in OSA patients. These future research directions have the potential to deepen our understanding of the pathogenesis of OSA and offer more effective management and care for patients.

## 5. Conclusions

In this study, some metabolites, such as leucine and 3-dehydrocarnitine, increased the risk of OSA, while gamma-glutamylvaline and betaine had protective effects. And several SCFA-producing gut microbial taxa were identified as potentially protective against OSA, a significant portion of which belong to the *Ruminococcaceae* family. Through bidirectional MR and MVMR, we provide evidence that gut microbiota and the derived metabolites are causally associated with OSA. These causally associated gut microbiota and metabolites are associated with disturbances in the gut microenvironment, changes in derived metabolites, and an increase in systemic pro-inflammatory response.

## Data Availability

The original contributions presented in the study are included in the article/Supplementary Materials, further inquiries can be directed to the corresponding authors.

## Supplementary Materials

The following supporting information can be downloaded at: https://www.mdpi.com/article/10.3390/nu15214544/s1. Additional File S1: Supplementary methods and results; Additional File S2: Supplementary Tables S1–S28; Additional File S3: Supplementary Figures S1–S12. References [75,76,77,78,79,80,81] are cited in the Supplementary Materials.
