# Supplementary material for: Causality Investigation between Gut Microbiota, Derived Metabolites, and Obstructive Sleep Apnea: A Bidirectional Mendelian Randomization Study
Source: Nutrients. 2023 Oct 26;15(21):4544. doi: 10.3390/nu15214544 (PMC10648878; doi:10.3390/nu15214544)
Supplement: Supplementary file 1 [file nutrients-15-04544-s001.zip › Additional File S1.pdf]

Additional File 1

Supplementary methods

Two-sample MR Analysis Supplementary methods

The IVW method combines individual MR estimates of SNPs to calculate a population-weighted estimate of potential causal effects [1]. To reduce the influence of heterogeneity on the results, a random effects IVW model was utilized as the main IVW method for the analysis.

MR-Egger is an adaptation of Egger regression. Egger regression, a method for detecting small-study bias in meta-analyses, can be used to evaluate bias from pleiotropy, and the slope coefficient of the Egger regression can be used for causal effect estimation. MR-Egger provides a sensitivity analysis for the robustness of the results in an MR study [2].

The WM method was used to combine data from multiple genetic variants into a single causal estimate. This estimator remains consistent even when up to 50% of the data are derived from invalid IVs [3].

Although the simple model is not as powerful as IVW, it can still be used as a complementary method for assessing the robustness of causality and pleiotropy [4]. In addition, weighted mode estimation is sensitive to challenging bandwidth selections [5].

MVMR Analysis Supplementary methods

GWAS summary data on obesity were obtained from the MRC-IEU Consortium (IEU GWAS ID: ukb-b-15541) [6]. GWAS summary data on sex (IEU GWAS ID: ukb-d-is_female) and smoking (IEU GWAS ID: ukb-d-22506_111) were obtained from the Neale Lab project [7]. To further eliminate the interaction between distinct exposures and avoid multicollinearity, the MVMR analysis was conducted separately for each common risk factor.

Pleiotropy and Sensitivity Analysis Supplementary methods

Any SNPs with potential pleiotropy were removed using MR-PRESSO, and MR analysis was subsequently reperformed to evaluate their significance and robustness.

The statistical strength of the IVs was assessed by calculating the F-statistic [4] using the following formula: $F = Beta^2/SE^2$. No significant weak IV effect was observed when the F-statistic was greater than 10.

reference

1. Burgess, S., F. Dudbridge, and S.G. Thompson, Combining information on multiple instrumental variables in Mendelian randomization: comparison of allele score and summarized data methods. Stat Med, 2016. 35(11): p. 1880-906.

2. Bowden, J., G. Davey Smith, and S. Burgess, Mendelian randomization with invalid instruments: effect estimation and bias detection through Egger regression. Int J Epidemiol, 2015. 44(2): p. 512-25.

3. Bowden, J., et al., Consistent Estimation in Mendelian Randomization with Some Invalid Instruments Using a Weighted Median Estimator. Genet Epidemiol, 2016. 40(4): p. 304-14.

4. Chen, W., et al., Causal Effect of Obstructive Sleep Apnea on Atrial Fibrillation: A Mendelian Randomization Study. J Am Heart Assoc, 2021. 10(23): p. e022560.

5. Manousaki, D., et al., Vitamin D levels and risk of type 1 diabetes: A Mendelian randomization study. PLoS Med, 2021. 18(2): p. e1003536.

6. Hemani, G., et al., The MR-Base platform supports systematic causal inference across the human phenome. Elife, 2018. 7.

7. Zheng, H., et al., Modifiable factors for migraine prophylaxis: A mendelian randomization analysis. Front Pharmacol, 2023. 14: p. 1010996.

Supplementary results

MVMR Analysis for OSA and Gut Microbiota/Metabolites

After correcting for obesity, male sex, or smoking, which are known risk factors for OSA, MVMR analysis revealed that some gut microbiota and metabolites were independently associated with OSA (Additional file 2: Tables S27 and S28). For example, when adjusted for obesity *family_Ruminococcaceae* (adjusted OR = 0.90, 95% CI: 0.81 to 1.00, P_{Robust} = 0.0426), *genus_Coprococcus2* (adjusted OR = 0.86, 95% CI: 0.77 to 0.95, P_{LASSO} = 0.0031), *genus_Eggerthella* (adjusted OR = 0.94, 95% CI: 0.90 to 0.99, P_{Robust} = 0.0237), and *genus_Eubacterium_xylanophilum_group* (adjusted OR = 0.89, 95% CI: 0.81 to 0.98, P_{Robust} = 0.0223) demonstrated significant and independent negative correlations with the occurrence of OSA. Even after adjusting for gender and smoke, these associations were also robust (Additional file 3: Figure 5A).

The *genus_Ruminococcaceae_UCG009* and *genus_Subdoligranulum* were found to be independently associated with an increased risk of OSA. MVMR analysis indicated that *genus_Subdoligranulum* was a potential independent risk factor for OSA, when adjusted for obesity (adjusted OR = 1.15, 95% CI: 1.02 to 1.29, P_{LASSO} = 0.0179), gender (adjusted OR = 1.10, 95% CI: 1.00 to 1.21, P_{Robust} = 0.0430), or smoke (adjusted OR = 1.10, 95% CI: 1.00 to 1.21, P_{IVW} = 0.0396). In the multivariable MR models, the results of the associations between *genus_Ruminococcaceae_UCG009* and OSA was also robust, when adjusted for obesity (adjusted OR = 1.10, 95% CI: 1.01 to 1.19, P_{Robust} = 0.0293; adjusted OR = 1.12, 95% CI: 1.01 to 1.24, P_{Median} = 0.0312; adjusted OR = 1.09, 95% CI: 1.02 to 1.17, P_{LASSO} = 0.0163), gender (adjusted OR = 1.07, 95% CI: 1.01 to 1.12, P_{Robust} = 0.0142), or smoke (adjusted OR = 1.08, 95% CI: 1.02 to 1.15, P_{Robust} = 0.0106) (Additional file 3: Figure 5A).

Alternatively, when metabolites were used as exposures, 3-dehydrocarnitine, epiandrosterone sulfate, and leucine were found to be potentially independent risk factors for the development of OSA (Additional file 3: Figure 5B and Additional file 2: Table S28). For example, epiandrosterone sulfate was a potential independent risk factor for OSA in the multivariable MR models, when adjusted for obesity (adjusted OR = 1.19, 95% CI: 1.10 to 1.28, $P_{\text{Robust}} = 1.44 \times 10^{-5}$; adjusted OR = 1.18, 95% CI: 1.03 to 1.36, $P_{\text{Median}} = 0.0184$; adjusted OR = 1.18, 95% CI: 1.04 to 1.33, $P_{\text{LASSO}} = 0.0081$), gender (adjusted OR = 1.23, 95% CI: 1.13 to 1.33, $P_{\text{Robust}} = 5.13 \times 10^{-7}$), or smoke (adjusted OR = 1.21, 95% CI: 1.12 to 1.31, $P_{\text{Robust}} = 1.29 \times 10^{-6}$).
