# Supplementary material for: Causality Investigation between Gut Microbiota, Derived Metabolites, and Obstructive Sleep Apnea: A Bidirectional Mendelian Randomization Study
Source: Nutrients. 2023 Oct 26;15(21):4544. doi: 10.3390/nu15214544 (PMC10648878; doi:10.3390/nu15214544)
Supplement: Supplementary file 1 [file nutrients-15-04544-s001.zip › Additional File S3.pdf]

Additional File 3: Supplementary Figures

Figure S1 The scatter plot of MR analysis between gut microbiota and OSA.

Figure S2 The scatter plot of MR analysis between OSA and gut microbiota.

Figure S3 The funnel plot of IVs in the MR analysis between gut microbiota and OSA.

Figure S4 The funnel plot of IVs in the MR analysis between OSA and gut microbiota.

Figure S5 The results of leave-one-out analysis between gut microbiota and OSA.

Figure S6 The results of leave-one-out analysis between OSA and gut microbiota.

Figure S7 The scatter plot of MR analysis between gut microbiota metabolites and OSA.

Figure S8 The scatter plot of MR analysis between OSA and gut microbiota metabolites.

Figure S9 The funnel plot of IVs in the MR analysis between gut microbiota metabolites and OSA.

Figure S10 The funnel plot of IVs in the MR analysis between OSA and gut microbiota metabolites.

Figure S11 The results of leave-one-out analysis between gut microbiota metabolites and OSA.

Figure S12 The results of leave-one-out analysis between OSA and gut microbiota metabolites.

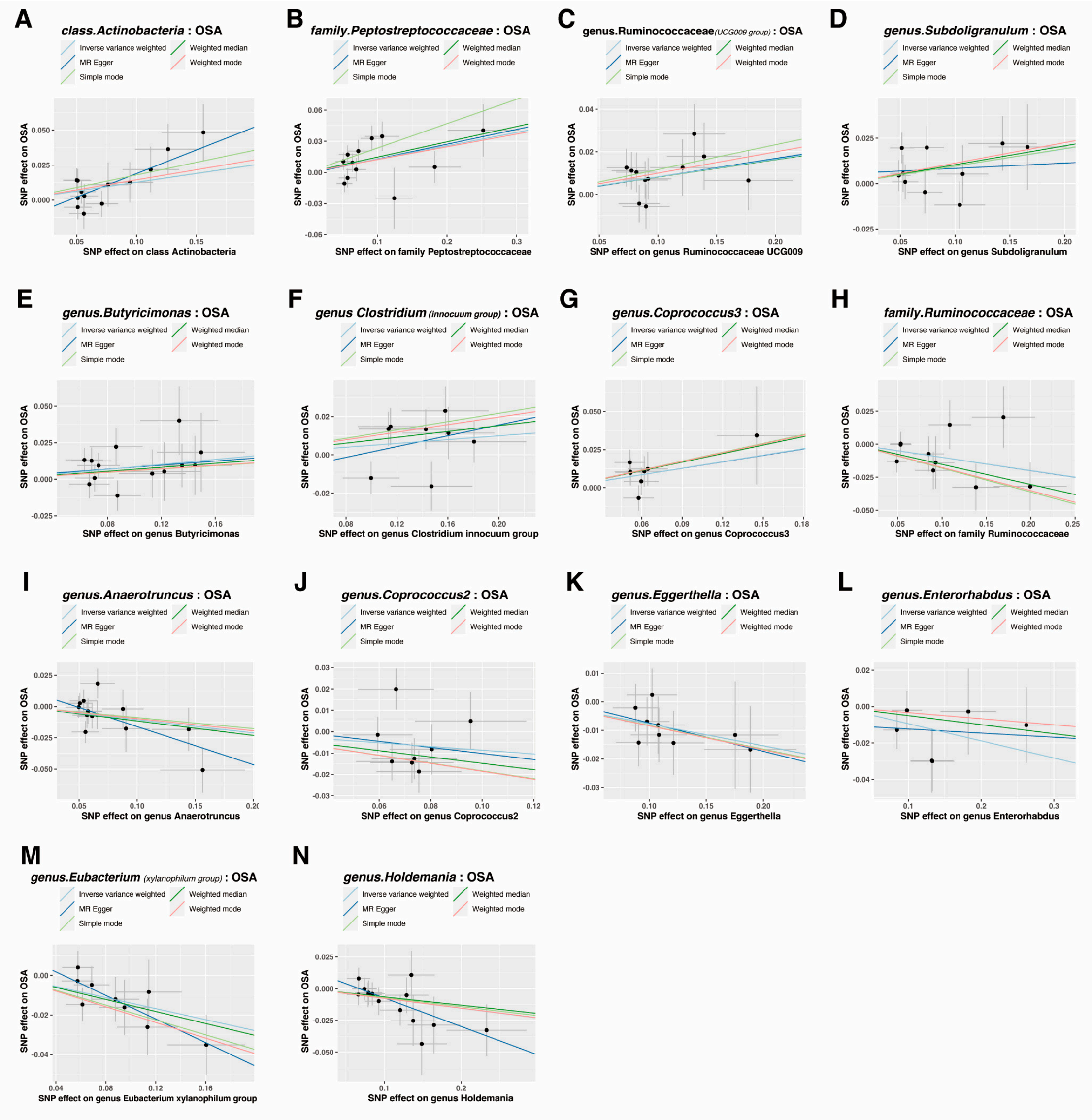

Figure S1 The scatter plot of MR analysis between gut microbiota and OSA.

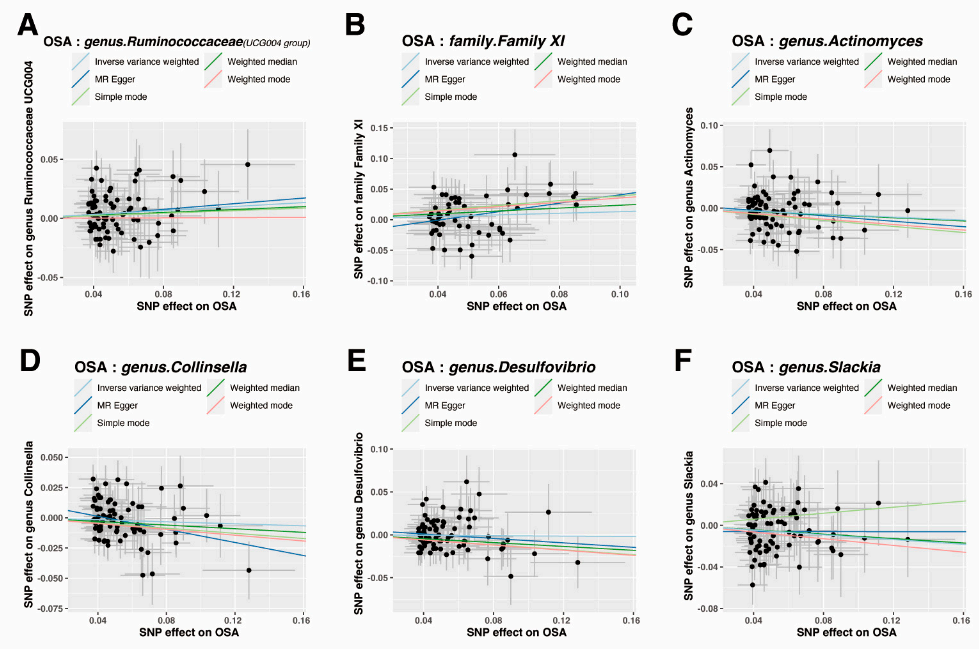

Figure S2 The scatter plot of MR analysis between OSA and gut microbiota.

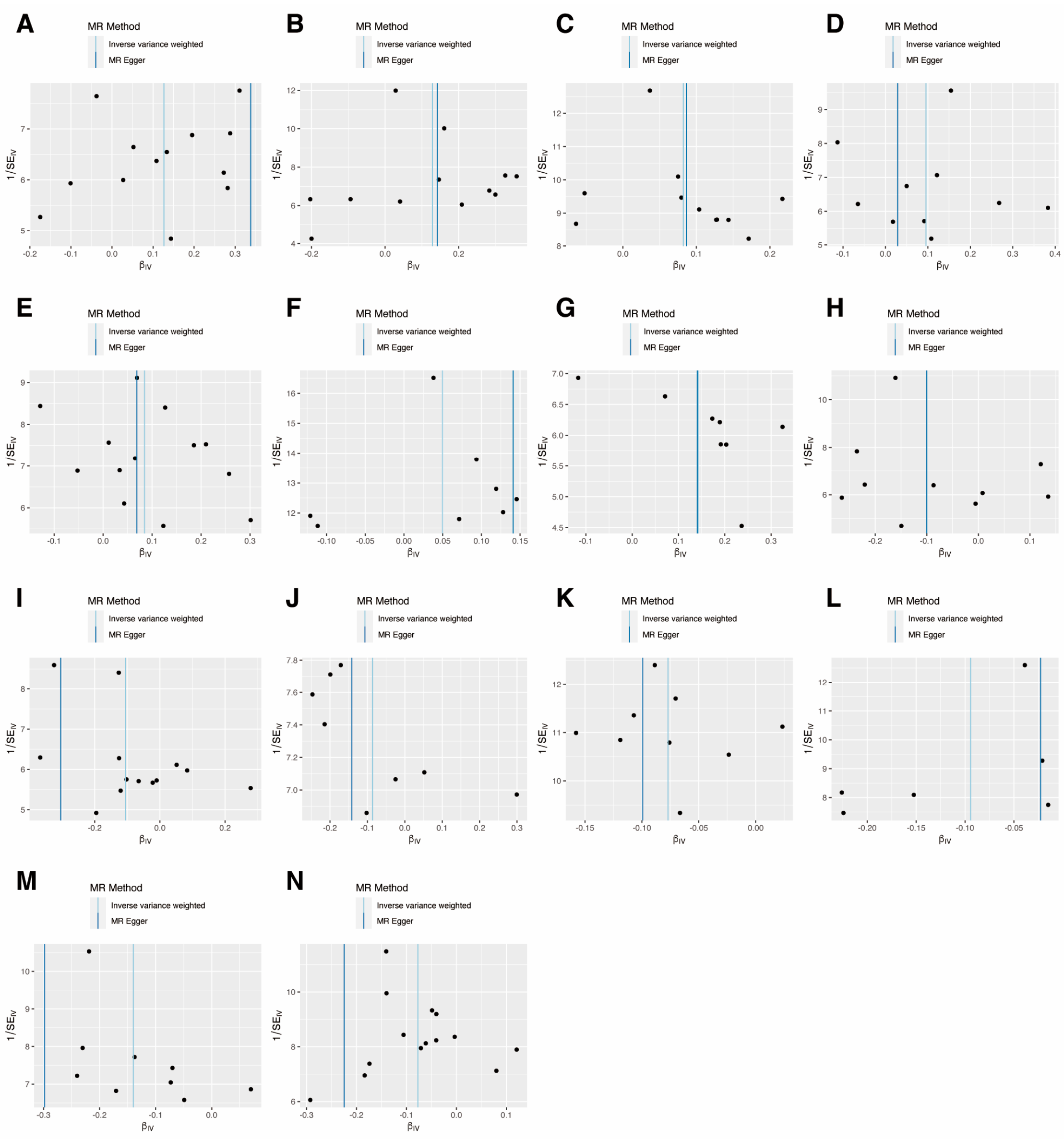

Figure S3 The funnel plot of IVs in the MR analysis between gut microbiota and OSA.

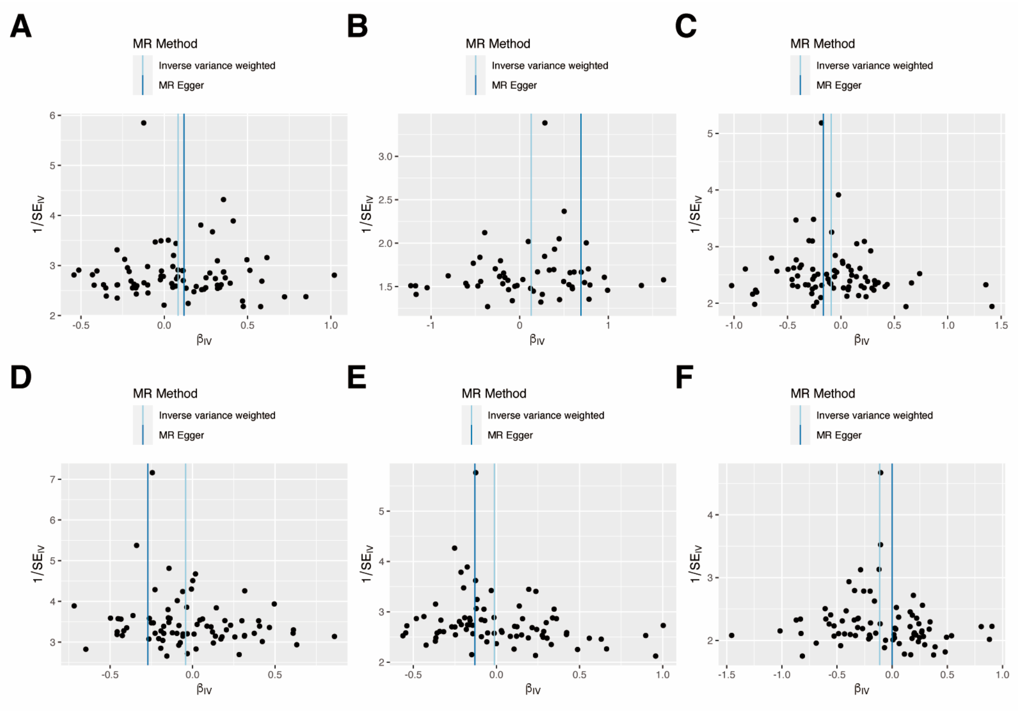

Figure S4 The funnel plot of IVs in the MR analysis between OSA and gut microbiota.

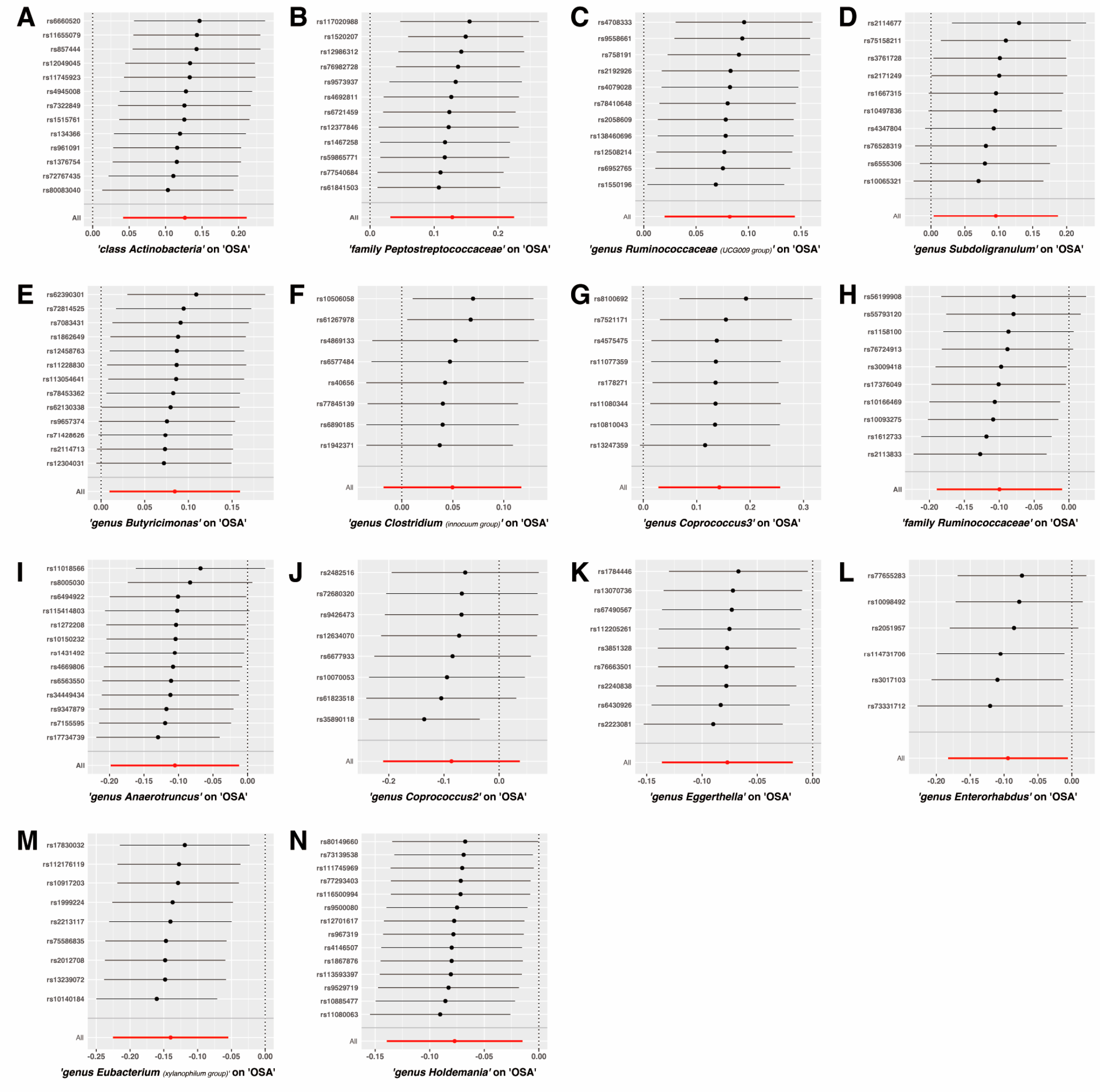

Figure S5 The results of leave-one-out analysis between gut microbiota and OSA.

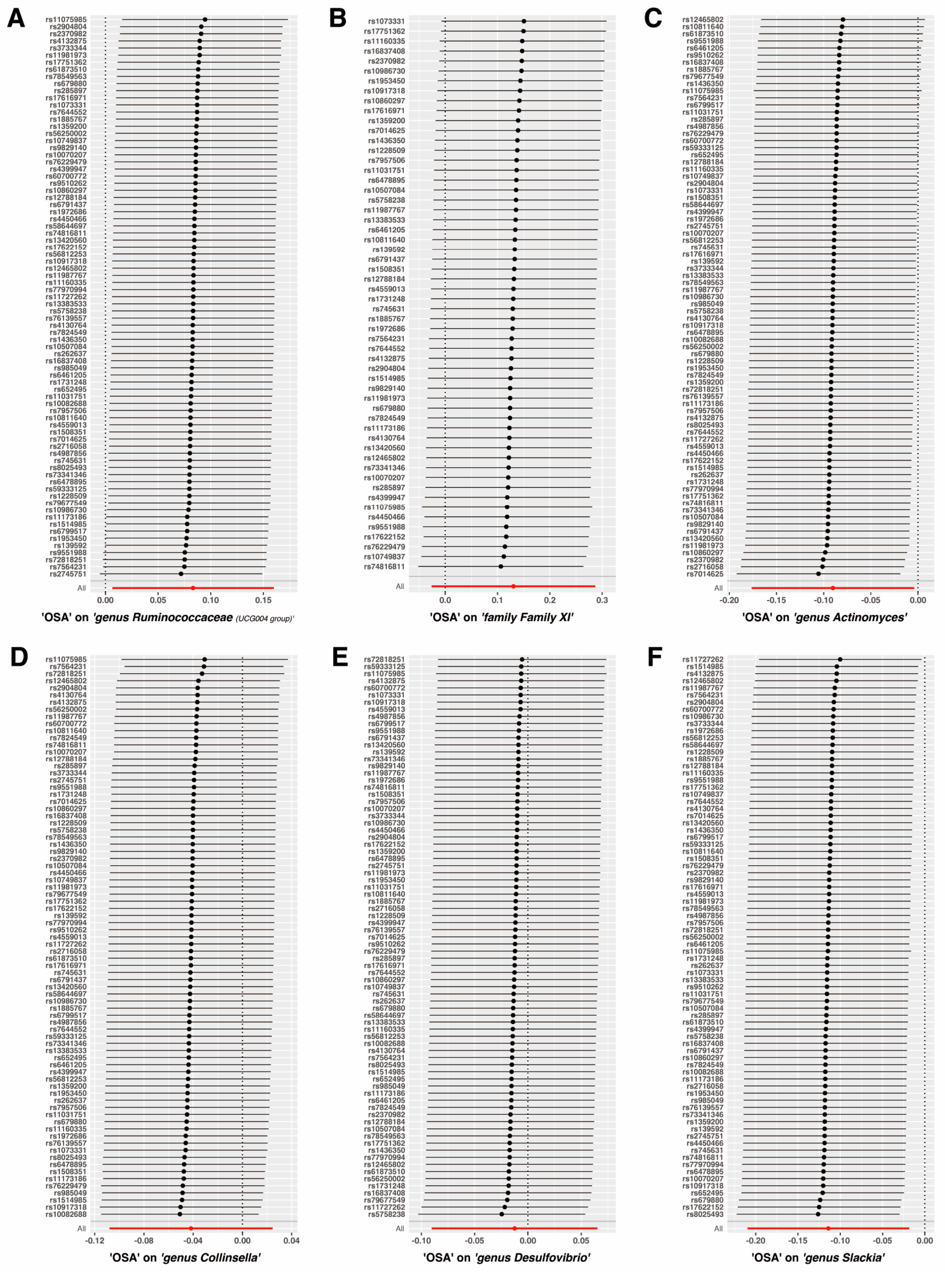

Figure S6 The results of leave-one-out analysis between OSA and gut microbiota.

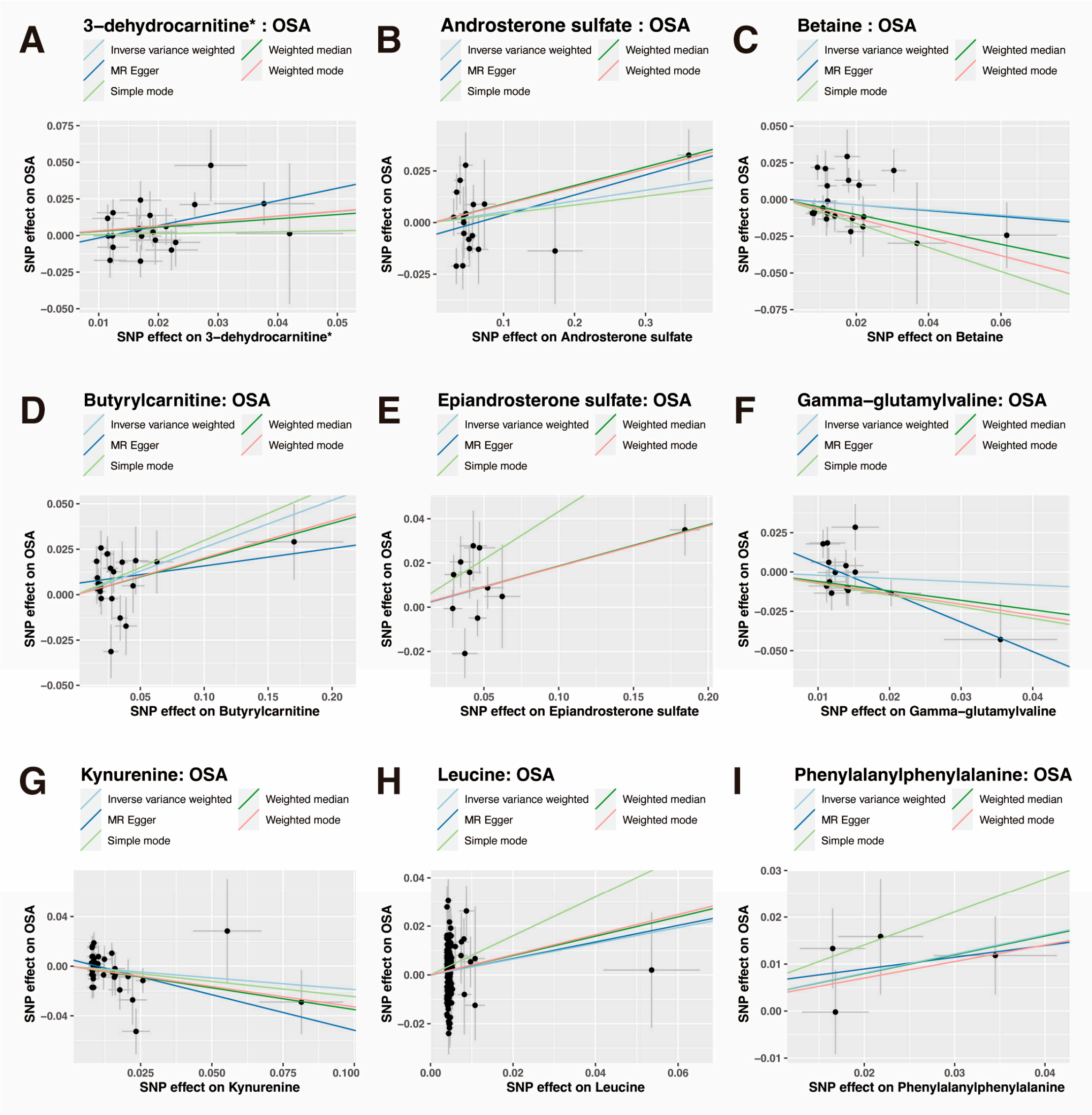

Figure S7 The scatter plot of MR analysis between gut microbiota metabolites and OSA.

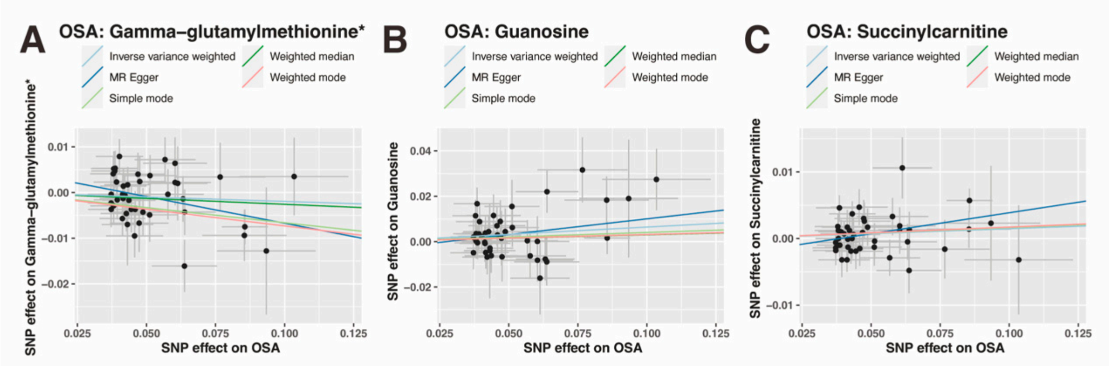

Figure S8 The scatter plot of MR analysis between OSA and gut microbiota metabolites.

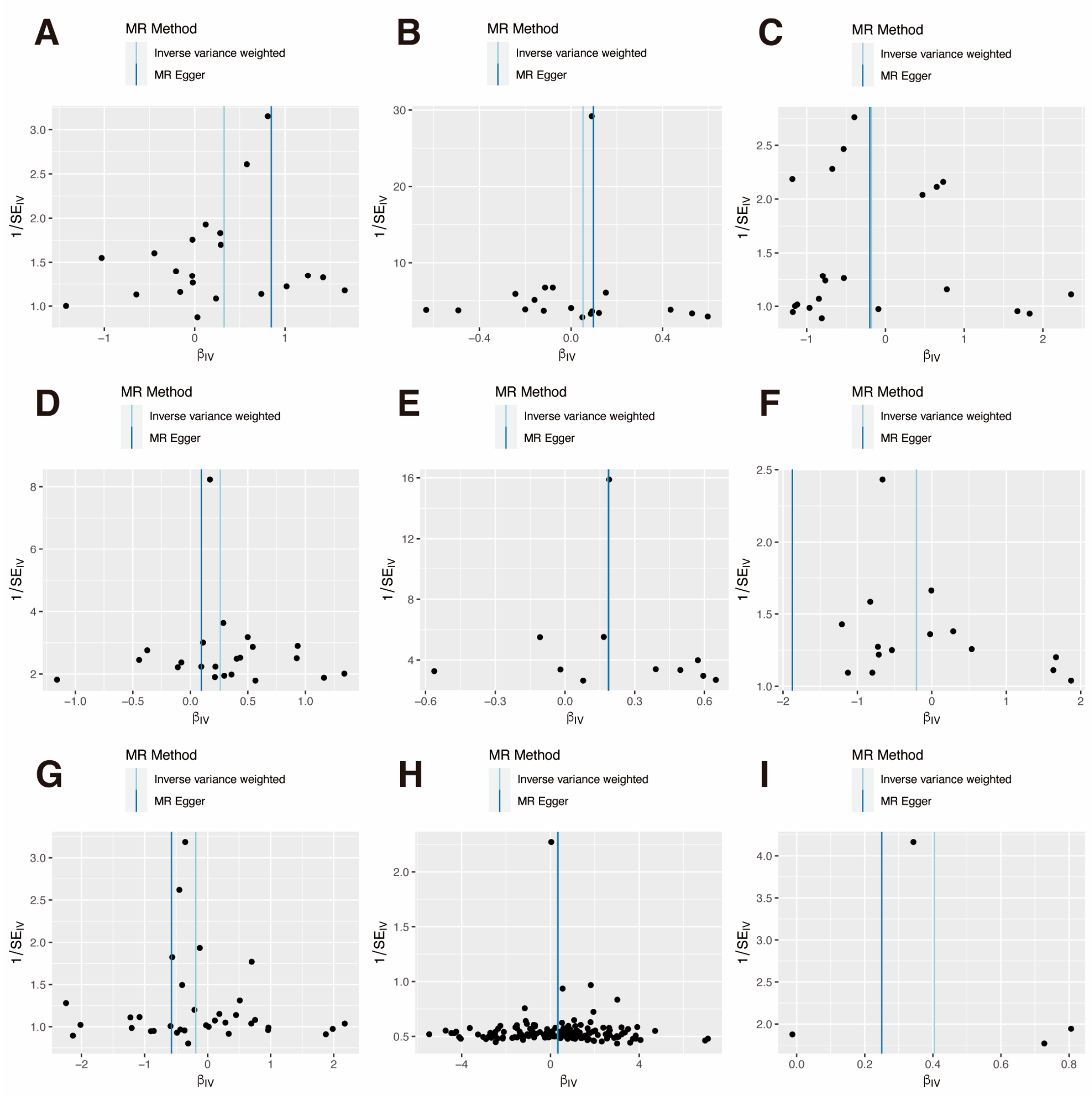

Figure S9 The funnel plot of IVs in the MR analysis between gut microbiota metabolites and OSA.

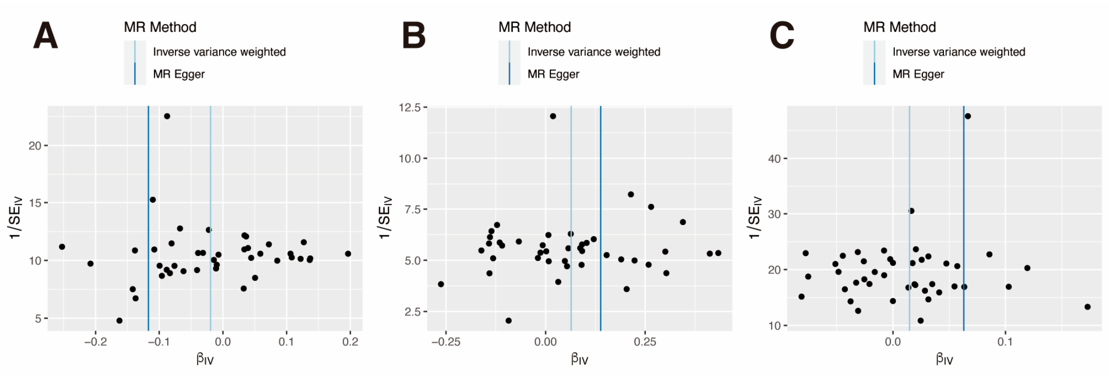

Figure S10 The funnel plot of IVs in the MR analysis between OSA and gut microbiota metabolites.

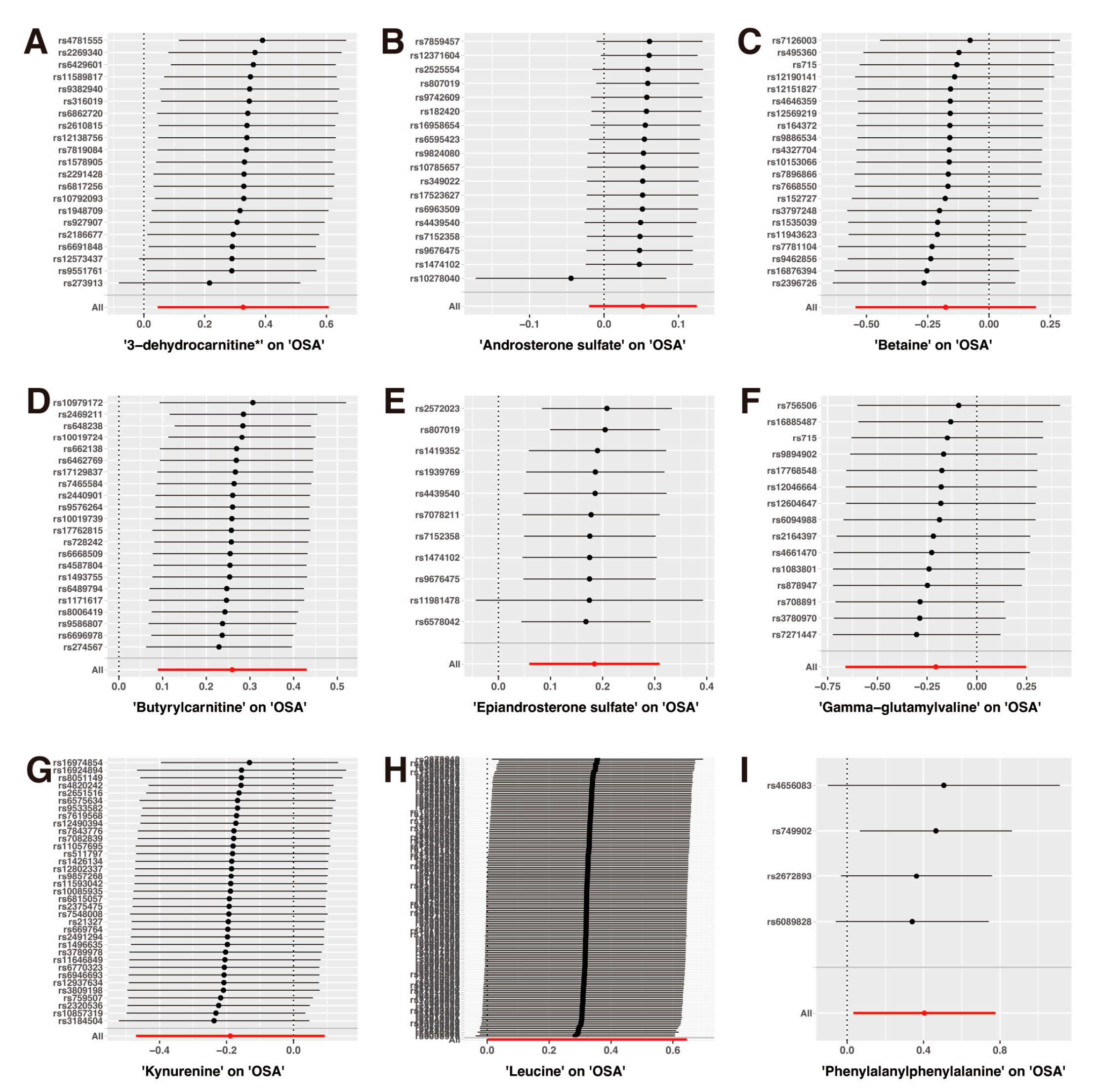

Figure S11 The results of leave-one-out analysis between gut microbiota metabolites and OSA.

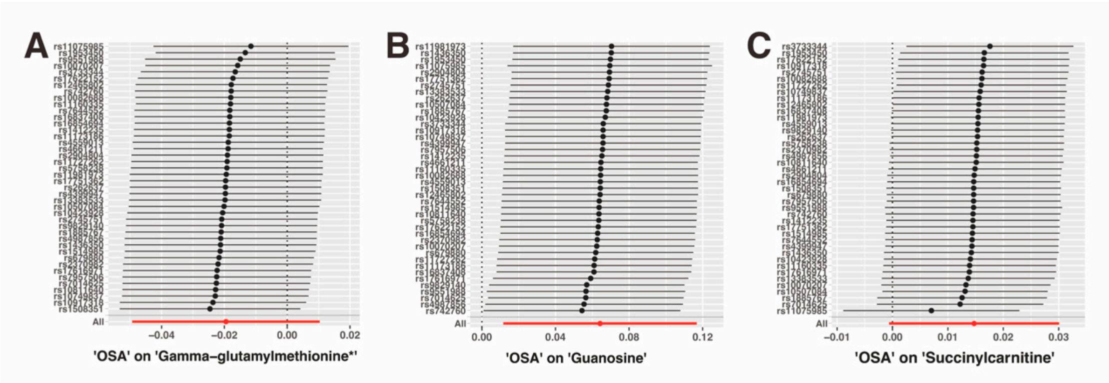

Figure S12 The results of leave-one-out analysis between OSA and gut microbiota metabolites.
